# Does My Step Look Big In This? A Visual Illusion Leads To Safer Stepping Behaviour

**DOI:** 10.1371/journal.pone.0004577

**Published:** 2009-02-25

**Authors:** David B. Elliott, Anna Vale, David Whitaker, John G. Buckley

**Affiliations:** 1 Bradford School of Optometry & Vision Science, University of Bradford, Bradford, West Yorkshire, United Kingdom; 2 School of Engineering, Design, & Technology, University of Bradford, Bradford, West Yorkshire, United Kingdom; University of California Davis, United States of America

## Abstract

**Background:**

Tripping is a common factor in falls and a typical safety strategy to avoid tripping on steps or stairs is to increase foot clearance over the step edge. In the present study we asked whether the perceived height of a step could be increased using a visual illusion and whether this would lead to the adoption of a safer stepping strategy, in terms of greater foot clearance over the step edge. The study also addressed the controversial question of whether motor actions are dissociated from visual perception.

**Methodology/Principal Findings:**

21 young, healthy subjects perceived the step to be higher in a configuration of the horizontal-vertical illusion compared to a reverse configuration (p = 0.01). During a simple stepping task, maximum toe elevation changed by an amount corresponding to the size of the visual illusion (p<0.001). Linear regression analyses showed highly significant associations between perceived step height and maximum toe elevation for all conditions.

**Conclusions/Significance:**

The perceived height of a step can be manipulated using a simple visual illusion, leading to the adoption of a safer stepping strategy in terms of greater foot clearance over a step edge. In addition, the strong link found between perception of a visual illusion and visuomotor action provides additional support to the view that the original, controversial proposal by Goodale and Milner (1992) of two separate and distinct visual streams for perception and visuomotor action should be re-evaluated.

## Introduction

The consequences of a fall for older adults are serious and the risk of injury, morbidity, and death from falling increases with age, with those over 75 being most vulnerable [reviewed in 1], [2]. In the UK, an estimated 2000 elderly people die every year as a result of a fall, with falls on stairs accounting for 60% of fall-related deaths [3]. Older adults are also more susceptible to serious injuries from falls, such as a broken hip or head injury, and are more likely than younger adults to be admitted to hospital or long stay institutions as a result of a fall [4]. The associated healthcare costs of older adult falls in Britain alone are estimated at around £1 billion per year [5]. Tripping, when the foot collides with an object causing loss of balance and either a stumble or fall, is a common factor in falls [6]. There are many reasons why elderly people are at a greater risk of tripping, [6] but one factor is that the elderly use variable and occasionally very small amounts of foot clearance on steps and stairs in both stair ascent [7], [8] and descent [9], [10], likely in an attempt to conserve energy. A typical safety strategy to avoid tripping on a step or stair, such as when blurred vision makes accurate judgement of the step height difficult, is to increase foot clearance over the step/stair edge in both stair ascent [7], [8] and descent [10]. In the present study we ask whether the perceived height of a step can be increased using a visual illusion and, more importantly, whether as a consequence this leads to the adoption of a safer stepping strategy, in terms of greater foot clearance over the step edge when stepping on to it.

It is far from obvious that a change in visual perception of a step's height should necessarily lead to a change in stepping strategy. Goodale and Milner [11] proposed a controversial, yet widely accepted hypothesis that the mediation of visual “perception” and visuomotor “action” are separated in the cortical visual system via the ventral and dorsal streams respectively. In support of this, Aglioti and colleagues [12] reported that the grasping or prehension action towards an object of illusory size (the Titchener circles/Ebbinghaus illusion) did not match their perceived size, but rather their actual size. Thus, whilst perception might be susceptible to visual (and other sensory) illusions, this need not be the case for motor action. Following this proposal, a large body of literature has arisen that has investigated the link between perceptual illusions and visuomotor actions, typically prehension and pointing [reviewed in 13], [14], [15]. Some studies report that perceptual changes do not lead to changes in action and thus support Aglioti's findings [14], whereas others report a direct link between perception and action [e.g. 16] and thus dispute Aglioti and colleagues' conclusions. Walking and stepping tasks have also resulted in conflicting reports of a link [17], [18] or a dissociation [19], [20] between action and perception of a visual illusion and a dissociation has been reported for the visual perception of the slope of a hill and motor-based actions indicating it's slant [21].

In the present study we show that a visual illusion, which induces a perceived increase in a step's riser height, results in increased safety during subsequent step negotiation because of an accompanying increase in toe elevation. These results demonstrate a simple, practical solution to reduce the likelihood of tripping when ascending a step or stairs.

## Results

**Figure 1:**
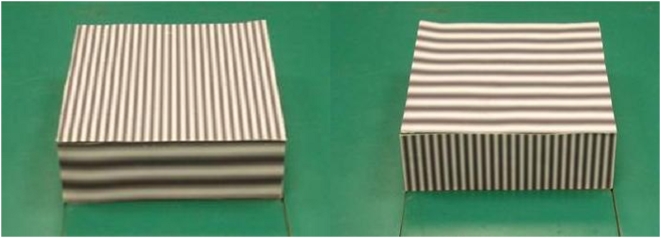
The two configurations of the step. On the left is the H configuration with Horizontal gratings on the step riser and on the right is the V configuration with Vertical gratings on the step riser.

**Figure 2:**
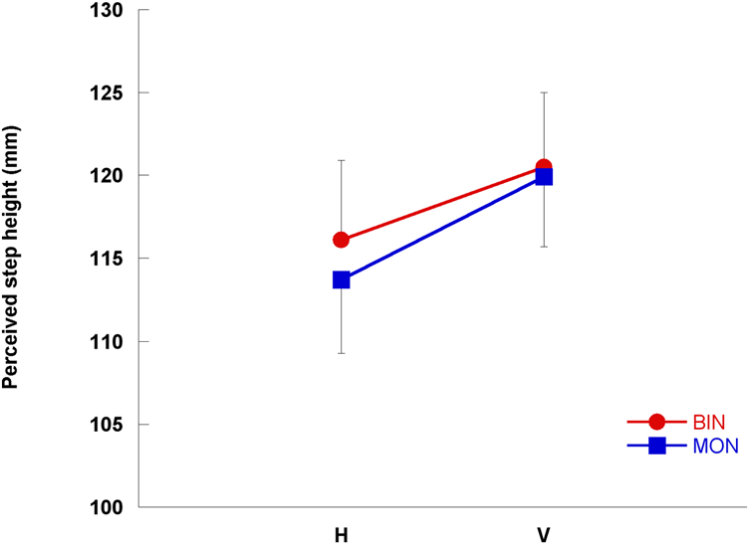
Perceived step height under different experimental conditions. Mean (±SE) perceived step height (mm) for H and V target configurations and monocular and binocular vision conditions.

Subjects perceived the step to be higher in the V configuration compared to the H configuration (Figure 1), increasing on average by 5.3 mm (∼4.5% of average perceived height, p = 0.01, Figure 2). There was no difference in the estimation of the height of the step between binocular and monocular vision conditions (p = 0.35).

**Figure 3:**
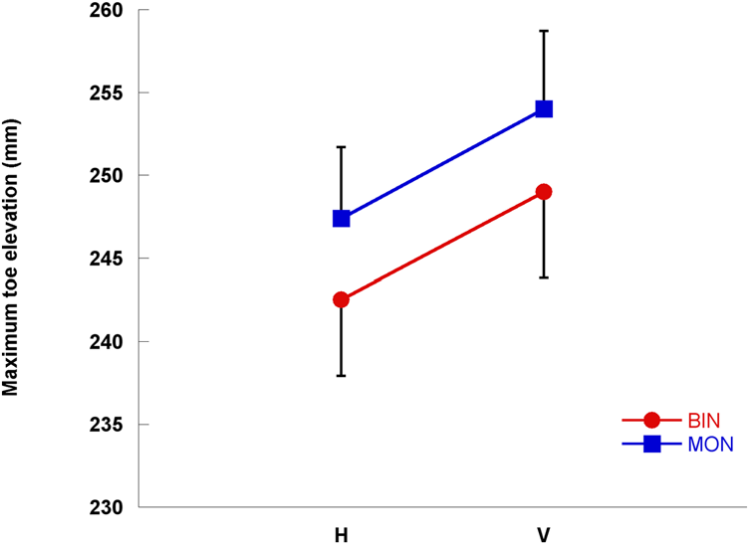
Maximum toe elevation under different experimental conditions. Mean (±SE) maximum lead toe elevation (mm) for H and V target configurations and monocular and binocular vision conditions.

Subjects increased maximum toe elevation in the V configuration compared to the H configuration (Figure 3, p<0.001) and toe elevation was greater for both configurations under monocular conditions (p = 0.003), but there was no significant interaction (p = 0.49). Toe elevation decreased with trial repetition (p = 0.001), but there were no significant interactions between repetition and monocular/binocular conditions (p = 0.17) or repetition and target (p = 0.37).

**Figure 4:**
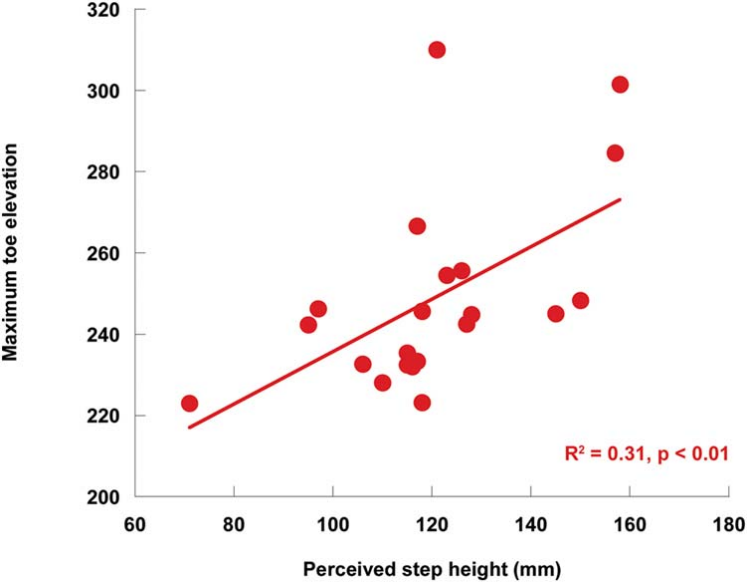
Perceived step height vs. maximum toe elevation. Scatterplot of perceived step height (mm) and maximum lead toe elevation (mm) in the V configuration and binocular vision condition.

The increased toe elevation led to increased lead-limb vertical toe clearance in the V configuration compared to the H configuration (p = 0.01). Linear regression analyses showed highly significant associations between perceived step height and maximum toe elevation in all conditions (V- Binocular R^2^ = 0.31, p<0.01, presented graphically in Figure 4; V- Monocular R^2^ = 0.30, p = 0.01; H- Binocular R^2^ = 0.36, p<0.01; H- Monocular R^2^ = 0.26, p = 0.02)

## Discussion

Perception of the height of a step was significantly affected by the configuration of the pattern superimposed on the step (figure 1), with the perceived step height being larger when the narrow, vertical sine wave gratings were placed on the step riser (figure 1, right panel). The 4.5% magnitude of the induced perceptual illusion was rather small in comparison to some configurations of the horizontal-vertical illusion that produce effect sizes of 10–20% [22]. This is most likely the result of the multifactorial nature of our illusory effect, with some factors accentuating the overall magnitude of the illusion, yet others negating it. For example, the effects of the Helmholtz square illusion [23] which are likely included in the V-H illusion used in the present study, results in objects appearing to expand in a direction orthogonal to the striped texture within them – the opposite effect to that which we attempted to create. No doubt a systematic evaluation of the factors contributing to the illusion would reveal stimulus parameters that would optimise the magnitude of the illusion.

Moreover, although the increase in toe clearance (and perceived increase in step height) seems relatively small (5.2 and 6.2 mm respectively) toe clearance during stair negotiation is typically between 20 to 50 mm with standard deviations only slightly less [10], [24]. Given these relatively small safety margins, the effect of increasing perceived step height by 5 to 6 mm is significant, and is therefore likely to reflect a substantial improvement in safety.

Despite “dummy trials” using different step heights to limit the effectiveness of using somatosensory feedback from previous trials to determine step height, maximum toe elevation and subsequent toe clearance reduced with repetition. This learning effect is commonly found with repeated stepping trials [7], [8], [25]. However, there were no interaction effects between step configuration and repetition, which indicates that the learning effect had no bearing on the main outcome measures of the present study. Both Marotta et al. [26] and Otto-de Haart et al. [27] suggested, based on their interpretation of Goodale and Milner's [11] two channel theory, that binocular conditions should provide perception-action dissociation but monocular conditions should not. However, our results demonstrate very similar effects of the horizontal-vertical illusion on perceived step height and toe elevation (figures 2 and 3) under binocular and monocular vision. Under monocular conditions there was an increase in toe elevation irrespective of step configuration, which is a straightforward precautionary measure unrelated to perceived step height and likely due to the loss of stereoscopic information about the step location under monocular conditions [25], [28].

When subjects stepped onto the step they lifted their lead foot higher in the V configuration condition compared to the H configuration condition as indicated by an increase in the maximum toe elevation (figure 3) and the amount of increase in the maximum toe elevation was similar to the perceived step height (figure 4). The results suggest that the changed perception of step height produced by the horizontal-vertical illusion led to a similar change in action by the lead foot to ensure that the step wasn't hit to avoid tripping and falling.

The strong link between visual perception and visuomotor action found in the present study is obviously at odds with those that have reported a clear dissociation between perception and action [early studies reviewed by 14], [ 19], [ see also 29], [ 30]–[32]. However, several studies have failed to replicate this dissociation between perception and action [early studies reviewed by 15], [18], [28], [see also 33], [34], and subsequent to Agiloti's original paper, it has been suggested that dissociation is only found under certain experimental conditions as highlighted below. The experimental design of this study was strongly in favour of finding a dissociation between perception and action in that:

- The perceptual size judgements were absolute rather than relative [35].
- Targets were real objects and not 2-D or virtual stimuli [14], [35].
- The action was ‘actual’ rather than ‘mimed’ [e.g. estimating perceived size of a target using a visuomotor task such as the distance between thumb and finger], [13, 15].
- The action was performed in real time [14], [19], [32] and in closed-loop conditions [e.g. 18], [35], [e.g. 36].
- Egocentric or observer-relative encoding of visuomotor actions was used [13], [20], [36].
- The target provided some cues to the peripheral visual system [14].
- The visuomotor task was repeated and learning was possible [35].
- The task was performed under binocular as well as monocular conditions [26], [27].
- The actions were highly practised and used the preferred foot (and preferably with the right hand [30])
- The locus of size illusions is deep within the ventral stream beyond the primary visual cortex V1 [31].

However, despite our experimental conditions strongly favouring finding a dissociation between perception and action, a clear link was found in that action followed perception. The number of conditions reported as exceptions to the rule that perception and action are dissociated are steadily increasing, which surely casts doubt on the original proposal. In addition, some studies have reported being unable to replicate previously reported dissociations once important control conditions were included [16], [37], [38]. It is possible that stepping tasks are processed in a very different way to other visuomotor tasks such as prehension, although earlier studies have suggested that a common visuomotor system likely subserves both upper and lower limb movements [18], [19].

One explanation for the dissociation between perception and action in prehension tasks could be that on-line visual feedback of hand position may lead to resistance of hand movements to visual illusions under closed-loop conditions [39]. For example, during tasks involving prehension, pointing and stepping to the end of a line under closed-loop conditions [12], [27], [40], [41], subjects may have used on-line visual feedback (e.g. cues of hand/foot position relative to target) to continuously ‘fine-tune’ grip size, finger position or foot placement, so that visual illusions have little or no effect on the final outcome of the action. Indeed, in experiments which include open-loop conditions, in which the subject inspects the target, but then closes their eyes for the remainder of the action task, then the effect of the visual illusion on the motor response often matches that of the perceptual response, and no dissociation between perception and action is found [18], [32], [36]. This suggests that using vision control in an on-line rather than a feed-forward manner is the key factor in determining a dissociation between perception and action tasks. On this point, it should be noted that some authors have interpreted such findings in a different way to allow their results to fit into the Goodale and Milner [11] two-stream hypothesis by suggesting that the dorsal stream lacks a memory of its own and must rely on the memory of the illusion-prone ventral stream as highlighted previously by Dassonville and Bala [33]. In this way open-loop conditions cause subjects to execute actions using an allocentric frame of reference via the memory of the ventral stream, and they argue that it is this that causes actions to be influenced by visual illusions [19], [32], [36]. In the present study, the locomotor task was performed in real time in closed-loop conditions and allowed on-line control, in that subjects could use visual feedback throughout the trial. However, although on-line visual feedback is used when intended foot placement changes during a step [42] in more standard stepping conditions, gaze is typically directed one or two steps ahead[43], [44]. Thus in the present study it is unlikely that on-line visual feedback would have been used to ‘fine tune’ toe clearance and instead margins of clearance would have been a consequence of uncertainty in determining step height during the approach. This lack of ‘fine tuning’ likely explains why we found both perception and action to be affected by the illusion. These results and others [e.g. 16], [18], [e.g. 33], [34] thus question the original, controversial proposal by Goodale and Milner [11] of two separate and distinct visual streams for visual perception and visuomotor action. The most parsimonious explanation of our results is that visuomotor actions are directed by the visual system without the need to invoke two wholly separate pathways for action and perception in the dorsal and ventral streams respectively.

To summarise, our results indicate that a visual illusion affected the perception of step riser height. During subsequent negotiation of the step when stepping on to it, the foot was lifted higher by a corresponding amount, and foot clearance was greater. This could have functional value in making the most dangerous steps, the first and last ones that most people trip over when ascending stairs [45], appear taller and generate a higher clearance and such an application deserves further study. In particular, stimulus parameters that would optimise the magnitude of the illusion for stepping up, while at the same time having no adverse safety effects when descending stairs, need to be determined. In addition, the effect of a visual illusion on toe clearance in a multiple step situation needs to be determined.

## Methods

### Ethics statement

The tenets of Declaration of Helsinki were followed and the study had approval of the University of Bradford Ethics Committee, with written informed consent being obtained from all participants.

### Subjects

Twenty one subjects (10 males and 11 females, mean age 28.2±8 years; height 169±12 cm; mass, 65.3±12.2 kg) were recruited from the University student population. Subjects were excluded from the study if they had any history of neurological, musculoskeletal or cardiovascular disorders that could affect their balance or gait, or had a history of eye disorders including amblyopia, strabismus or congenital cataract. All subjects had good visual acuity (better than 0.1 logMAR, Snellen equivalent 6/7.5) in both eyes and good depth perception (60 seconds of arc or better on the TNO stereoacuity test).

### Target

The perceptual illusion was produced by superimposing visual patterns onto a step (W464×L508 mm×H152 mm) in one of two configurations. A high contrast vertical sine wave grating with relatively high spatial frequency (54 cycles per metre) was placed on the front face (the riser) of the step with a horizontal grating of relative low spatial frequency (20 cycles per metre) on the top surface of the step (figure 1, right panel). This was termed the V configuration. The second configuration (H) was the inverse of this, i.e. the horizontal grating was placed on the riser (figure 1, left panel). These patterns introduce a version of the horizontal-vertical illusion [22] in which vertically-oriented lines appear longer than horizontal. The existence of the effect (if not its underlying biological cause) is well known and vertical stripes are widely used in the fashion industry to enhance perceived height and slenderness. The variation in spatial frequency also induces a type of size-contrast illusion in which the perceived size of an object (in this case the step) is judged relative to the size of texture either within or surrounding the object [23]. Fine texture leads to an overestimation of object size, with the reverse effect for coarse texture. The effective height of the step from the point of the subject's eye was 110 cm (step height of 152 mm viewed from a mean two walking steps distance of 140 cm and mean height of 169 cm).

### Protocol

Perceived step height was measured with the subject situated two walking paces away from the step's leading edge (mean distance 1.40±0.20 m) by the experimenter holding a 0–300 mm sliding scale in the same plane as the step but at head height. A Bekesy staircase method was used in which the scale was increased and decreased in size until the subject indicated it matched the perceived height of the step. An individual two walking paces distance was chosen as this is how far ahead subjects typically look when required to step over an obstacle in their travel path during locomotion [43], [44]. Measurements were taken for four conditions: monocularly and binocularly for both the H and V configurations of the step, using a randomised order of testing. The dominant eye, as determined by the Kay Dominance Eye test, was chosen for the monocular condition with the other eye occluded. Measurements were made under monocular and binocular conditions because Marotta et al. [26] and Otto-de Haart et al. [27] have suggested that binocular conditions should provide perception-action dissociation but monocular conditions should not according to their interpretation of Goodale and Milner's two channel theory [11].

Once perceived step height was measured for all conditions, subjects completed repeated stepping trials. Each trial consisted of the subject walking up to the step from two walking pace lengths away and then stepping onto it. A member of the research team was positioned near the front edge of the step to ensure that if subjects should trip or stumble they didn't fall. Subjects wore their own flat shoes and used a self-selected lead limb throughout the trails. They also used their habitual refractive correction and kept their eyes open throughout the trial meaning that data were collected in closed-loop conditions. The laboratory was well lit with an ambient illuminance of 400 lux. Stepping trials were made in monocular and binocular conditions and for both the V and H configurations of the step and each trial was repeated five times in random order, giving a total of 20 stepping measurements for each subject. In addition, six “dummy trials” were included, where the height of the step was randomly adjusted by −10 mm or +5 mm every third trial to limit the effectiveness of using somatosensory feedback from previous trials to estimate the height of the step. No data were collected during these trials and subjects were advised that the height of the step would be varied throughout the study.

Three-dimensional lower limb segmental kinematic data of the stepping action were collected (at 100 Hz) using an eight-camera, motion capture system (Vicon MX; Oxford Metrics Ltd, Oxford, UK). Reflective markers (6 and 14 mm diameter) were attached either directly onto the skin or shoes in the following locations: superior aspects of the 2^nd^ and 5^th^ metatarsal heads, end of 2^nd^ toes, lateral malleoli and posterior aspect of the calcenai. Markers were also placed on the sternum, and on the upper front edge of the step to determine its location and height within the laboratory coordinate system. A virtual marker, representing the inferior tip of the shoe (virtual shoe tip) was determined by reconstructing its position relative to the markers placed on the 2^nd^ and 5^th^ metatarsal heads and end of 2^nd^ toe. The 3D coordinate data of the sternum marker, each foot marker (including the virtual shoe tip), and the markers placed on the raised surface were exported in ASCII format for further analysis. It has been suggested that the central nervous system ensures adequate foot clearance over a step by controlling maximum toe elevation [46], which was therefore the primary visuomotor action assessed. More details regarding the measurement of the gait/stepping parameters analysed can be found in earlier reports [24], [47].
